# Plasma Parameters and Etching Characteristics of SiO_x_N_y_ Films in CF_4_ + O_2_ + X (X = C_4_F_8_ or CF_2_Br_2_) Gas Mixtures

**DOI:** 10.3390/ma13235476

**Published:** 2020-12-01

**Authors:** Yunho Nam, Alexander Efremov, Byung Jun Lee, Kwang-Ho Kwon

**Affiliations:** 1 Department of Control and Instrumentation Engineering, Korea University, Sejong 30019, Korea; yhnam09@korea.ac.kr (Y.N.); byung_jun@korea.ac.kr (B.J.L.); 2 Department of Electronic Devices & Materials Technology, State University of Chemistry & Technology, 7 Sheremetevsky av, 153000 Ivanovo, Russia; amefremov@yandex.ru

**Keywords:** global warming potential, SiO_x_N_y_ etching rate, plasma diagnostics, reaction kinetics, polymerization

## Abstract

In this work, we carried out the study of CF_4_ + O_2_ + X (X = C_4_F_8_ or CF_2_Br_2_) gas chemistries in respect to the SiO_x_N_y_ reactive-ion etching process in a low power regime. The interest in the liquid CF_2_Br_2_ as an additive component is motivated by its generally unknown plasma etching performance. The combination of various diagnostic tools (double Langmuir probe, quadrupole mass-spectrometry, X-ray photoelectron spectroscopy) allowed us to compare the effects of CF_4_/X mixing ratio, input power and gas pressure on gas-phase plasma characteristics as well as to analyze the SiO_x_N_y_ etching kinetics in terms of process-condition-dependent effective reaction probability. It was found that the given gas systems are characterized by: (1) similar changes in plasma parameters (electron temperature, ion current density) and fluxes of active species with variations in processing conditions; (2) identical behaviors of SiO_x_N_y_ etching rates, as determined by the neutral-flux-limited process regime; and (3) non-constant SiO_x_N_y_ + F reaction probabilities due to changes in the polymer deposition/removal balance. The features of CF_4_ + CF_2_Br_2_ + O_2_ plasma are lower polymerization ability (due to the lower flux of CF_x_ radicals) and a bit more vertical etching profile (due to the lower neutral/charged ratio).

## 1. Introduction

Silicon dioxide (SiO_2_) and silicon nitride (Si_3_N_4_) are two indispensable dielectric materials traditionally used as gate dielectrics in field-effect structures, hard masks for patterning of various functional layers, spacer insulators as well as passivation and protective coatings in micro- and nano-electronic devices [1,2,3]. The silicon oxynitride (SiO_x_N_y_) also exhibits a dielectric nature and was found to be a perspective material for photo-electronic device applications. Particularly, it is characterized by low optical loss (high optical transparency in the infra-red wavelength region) as well as by a wide-range refractive index which may be adjusted by the change in the O/N stoichiometry [4,5].

Since most of the above applications for silicon-bases dielectric materials require precision patterning of preliminary deposited continuous layers, the development of a suitable “dry” (plasma-assisted) etching process is an essential problem for obtaining both an accurate pattern transfer and desirable device parameters. In fact, this requires the understanding of etching mechanisms that allow one to adjust etching kinetics and output process parameters (etching rate, etching selectivity and anisotropy, surface damage and residues) through the deliberate choice of processing conditions. When summarizing the previous etching experience for SiO_2_ [6,7,8,9,10,11,12,13,14,15], Si_3_N_4_ [8,9,10,11,12,13,14,15] and SiO_x_N_y_ [16,17,18], the following conclusions can be made:

1) The patterning of these materials is normally provided by the C_x_F_y_ gas family (CF_4_, C_2_F_6_, C_3_F_6_, C_4_F_8_) under reactive-ion etching (RIE) conditions. The mixing of any fluorocarbon gas with Ar and/or O_2_ as well as the combination of two C_x_F_y_ components with different C/F ratios in one gas mixture allows the flexible adjustment of etching kinetics, residues and pattern profile through changes in both F atom density and deposition/removal balance for the fluorocarbon polymer film [8,11,12,13,14].
2) The heterogeneous reaction F + Si-O → Si-F + O has a sufficient energy threshold (as the Si-O bond of ~799 kJ/mol is stronger than the Si-F one of ~552 kJ/mol [19]) and cannot occur spontaneously at typical process temperatures. Accordingly, the dry etching of SiO_2_ requires ion bombardment which produces adsorption sites for F atoms (through the destruction of Si-O bonds) and cleans the surface from non-saturated SiF_x_ compounds [2,6,7,8,9,20]. Ion energies above 150–200 eV surely provide the reaction-rate-limited etching regime where the change in SiO_2_ etching rate follows the behavior of F atom density [2,6,21].
3) The heterogeneous reaction F + Si-N → Si-F + N is possible (as the Si-N bond of ~470 kJ/mol is weaker compared with the Si-F one [19]), but the Si_3_N_4_ etching rate exhibits an evident acceleration by ion bombardment [12]. The latter is probably due to the higher adsorption probability of etchant species on partially liberated silicon atoms. Accordingly, under conditions of the RIE process, Si_3_N_4_ also demonstrates the reaction-rate-limited etching regime [9,10,11,12] and is characterized by much higher absolute etching rates compared with SiO_2_ [11,12]. In addition, the thickness of the fluorocarbon polymer film on Si_3_N_4_ was found to be higher compared with SiO_2_ (that looks quite expectable for oxygen-free and oxygen-containing surfaces), but lower than that on Si [8,15]. The last effect is obviously connected with the lower sticking probability for polymerizing radicals [13].
4) Etching characteristics of SiO_x_N_y_ films has received much less attention compared with silicon dioxide and silicon nitride. From several published works, it can be understood that: (a) under typical RIE conditions, SiO_x_N_y_ etching kinetics is also controlled by the F atom density [22]; (b) an increase in O/N ratio expectably reduces the SiO_x_N_y_ etching rate [17,18], as this corresponds to the transition from Si_3_N_4_ to SiO_2_; and (c) the etching damage changes the O/N ratio on the treated surface and thus influences the refractive index in respect to the as-deposited film [16,17]. At the same time, existing studies do not provide a comparison of etching kinetics and mechanisms with different gas chemistries, including chemistries with two fluorocarbon components. Such a situation retards the development of the effective dry etching technology for SiO_x_N_y_ films.

It should be mentioned that the common problem of all fluorocarbon gases used in SiO_2_, Si_3_N_4_ and SiO_x_N_y_ reactive-ion etching processes is their high global warming potential (GWP) due to the destructive effect on the ozone layer [23]. Recently, environmental pollution and resource consumption have received increasing attention in the semiconductor industry, and several studies on the eco-friendly manufacturing processes have been conducted [24,25]. One way for improving the situation is to replace conventional high-GWP process chemistries with those that exhibit a reduced environmental impact [26,27]. That is why several low-GWP fluorocarbon compounds have been tested for application in plasma-assisted etching processes [28,29,30]. Among those, CF_2_Br_2_ shows a considerably low GWP index of 231 (compared with over 8000 for the conventional C_x_F_y_ family [23]) and exists in the liquid state at room temperature [31,32]. The latter means much easier trapping and/or recovery procedures compared with gaseous fluorocarbon compounds. At the same time, the etching performance of CF_2_Br_2_ in respect to silicon-based materials is generally not known. As such, the uncertain relationships between processing conditions, plasma parameters and etching kinetics are reasons for real perspectives of CF_2_Br_2_ to be a replacement for widely used gaseous fluorocarbons and thus, retard the transition to environmental-friendly dry etching technologies.

The main idea of the current study was to investigate the reactive-ion etching kinetics, etching mechanism and surface states for SiO_x_N_y_ films in CF_4_ + O_2_ + X (X = C_4_F_8_ or CF_2_Br_2_) inductively coupled plasmas under one and the same operation conditions. Accordingly, the main goals were: (1) to compare performances of high-GWP C_4_F_8_ and low-GWP CF_2_Br_2_ as additive components to adjust the SiO_x_N_y_ etching characteristics, such as etching rate, etching residues and etching profile; (2) to study interconnections between processing parameters (input power, gas pressure and CF_4_/X mixing ratios), plasma chemistry and gas-phase plasma characteristics (electron temperature, energy of ion bombardment, densities and fluxes of plasma active species); and (3) to analyze the SiO_x_N_y_ etching kinetics in order to formulate reasonable approaches on etching mechanisms in given gas systems. In addition, we focused our attention on the low power etching regime that produces at least ten times lower plasma density compared with the conventional reactive-ion etching process. According to Refs. [33,34], such a regime is characterized by both more anisotropic etching (probably, due to the lower neutral/charged ratio) and weaker surface damage because of reduced ion flux. The latter effect allows one to minimize the degradation of electric characteristics for dielectric materials. Since all these questions have not been studied yet, the corresponding data are expected to be quite useful for understanding features of SiO_x_N_y_ etching with CF_2_Br_2_ gas and thus, for the optimization of dry etching technology towards environmental-friendly processes.

## 2. Experimental Part and Data Analysis

### 2.1. Experimental Setup and Procedures

Both etching and plasma diagnostics experiments were performed in the planar inductively coupled plasma (ICP) (Homemade ICP etching system) reactor; the same as that used in our previous studies [6,22,35]. Cylindrical (*r* = 16 cm, *l* = 13 cm) reactor chamber was made from anodized aluminum. Plasma was excited using the 13.56 MHz power supply connected to the copper coil at the top of the chamber through the matching network. Another 12.56 MHz rf generator biased the bottom electrode (the substrate holder) to control the ion bombardment energy through the negative dc bias voltage $\left(-U_{dc}\right)$. The latter was measured using a high-voltage probe (AMN-CTR, Youngsin-RF Co., Ltd, Seoul, Korea) The bottom electrode had a built-in water-flow cooling system that allowed us to maintain its temperature $\left(T_{s}\right)$ at the nearly constant value of ~17 °C within the processing times τ~5 min. The variable process parameters were input power (=100–500 W), gas pressure (p = 4–10 mTorr) and the composition of CF_4_ + O_2_ + X (X = C_4_F_8_ or CF_2_Br_2_) feed gas. The latter was set by various partial flow rates for fluorocarbon components at fixed both O_2_ flow rate (4 sccm) and the total gas flow rate (44 sccm). Accordingly, the fraction of O_2_ in a feed gas, $y_{O_{2}}$, was always~ 10%, and the change in the partial flow rate for the X gas in the range of 0–40 sccm provided the full substitution of CF_4_ for C_4_F_8_ or CF_2_Br_2_ (i.e., to the transition between 90% CF_4_ + 10% O_2_ and 90% X + 10% O_2_ gas systems). In addition, the bias power ($W_{dc}$) was always kept at a constant level of 400 W. Such situation corresponded to the variable $-U_{dc}$ value, according to the dependence of positive ion flux on other processing conditions.

Plasma parameters and gas-phase composition were investigated using a combination of Langmuir probe diagnostics and quadrupole mass-spectrometry. The double Langmuir probe (DLP2000, Plasmart Inc, Deajeon, Korea) provided the information on electron temperature ($T_{e}$) and ion current density ($J_{+}$). The treatment of raw I–V curves was based on the well-known procedure described in Refs. [36,37]. In order to minimize experimental errors due to the deposition of fluorocarbon polymer on probe tips, the latter were cleaned in 50% Ar + 50% O_2_ plasma before and after each measurement. Our previous works have demonstrated the efficiency of such procedure to obtain adequate diagnostics results [22,35,38]. The residual gas analyzer (LEYSPEC view 200S, Leybold GmbH Inc, Cologne, Germany) with a quadruple filter was used to determine steady-state densities of neutral species in the plasma on regime. For this purpose, the gas was continuously collected directly from the reactor chamber and then, ionized by the 50 eV electron beam that allowed us to neglect the formation of multi-charge ions. Measurements were conducted in the range of 1–200 atomic mass units. The species of primary interest were F atoms (as the main chemical etchants) and CF_x_ (x = 1, 2) radicals (as the main polymerizing agents).

In order to study the etching kinetics for SiO_x_N_y_ films, the latter were deposited on Si (111) wafer using the plasma-enhanced chemical vapor deposition (PECVD) (AMAT, Santa Clara, CA, USA) method. The precursor gases were Si_3_H_4_, O_2_, and N_2_. The combination of substrate temperature of 400 °C and a deposition time of 15 min provided a uniform film with a thickness of ~1 μm. For etching experiments, we used the fragments of whole wafer with a size of about 2 × 2 cm which were placed at the middle part of the bottom electrode. The small sample size allowed us to neglect the loading effect as well as to provide the etching regime controlled by heterogeneous process kinetics. Preliminary experiments showed no principal differences in both raw I–V curves and related plasma parameters obtained with and without sample loading. Therefore, one can neglect the sensitivity of gas-phase plasma parameters to etching products as well as consider the gas phase to be the permanent source of active species. Etched depths (Δh) were determined using the surface profiler Alpha-Step 500 (Tencor, Milpitascity, CA, USA) for the processing time τ = 1 min. For this purpose, we developed a partial surface masking by the photoresist AZ1512 with a thickness of ~ 1.5 μm. The quasi-linear shape of Δh=f(τ)  curves in both gas systems allowed us to assume the steady-state etching kinetics as well as pointed out the very weak change in the sample temperature within given processing times. That is why we found the SiO_x_N_y_ etching rate simply as R=Δh/τ as well as ignored the sample temperature-related effects when analyzing the etching kinetics. The chemical compositions of plasma-treated SiO_x_N_y_ surfaces were examined using X-ray photoelectron spectroscopy (K-Alpha, Thermo VG, UK) with Mg K_α_ 1253.6 eV radiation operating at 260W. The binding energies were calibrated using C(1s) peak at 284.5eV.

In order to study the SiO_x_N_y_ etching profile, we prepared a hole-type pattern with a diameter of 500 nm using the α-Si hard mask. The mask layer with a thickness of ~250 nm was deposited over the SiO_x_N_y_ film by PECVD from Si_3_H_4_ atmosphere at 400 °C. The profiles of hole structures with equal depths (i.e., obtained after different processing times) in 45% CF_4_ + 45% C_4_F_8_ + 10% O_2_ and 45% CF_4_ + 45% CF_2_Br_2_ + 10% O_2_ plasmas were controlled by the field-emission scanning electron microscope (MIRA3 LMH, TESCAN).

### 2.2. Approaches for the Analysis of Etching Kinetics

For the phenomenological analysis of SiO_x_N_y_ etching kinetics, one can account for known features of the reactive-ion etching process for SiO_2_ and Si_3_N_4_ in fluorocarbon-based plasmas [13,14,15,22,35,38,39,40]. These are as follows:

1) The experimentally obtained etching rate R is composed of two summands, $R_{phys}+R_{chem}$. The first summand represents the rate of physical sputtering $Y_{S}\Gamma_{+}$ [38,39], where $Y_{S}$ ~ $\sqrt{\varepsilon_{i}}$ [39] is the sputtering yield, $\varepsilon_{i}=\left|-U_{f}-U_{dc}\right|$ is the ion bombardment energy, $-U_{f}\approx 0.5T_{e}\ln\left(m_{e}/2.3m_{i}\right)$ is the floating potential, and $\Gamma_{+}\approx J_{+}/e$ is the flux of positive ions. The second summand represents the rate of ion-assisted chemical reaction $\gamma_{R}\Gamma_{F}$ [22,35,38], where $\gamma_{R}=s_{0}\left(1-\theta \right)$ [22,35] is the effective reaction probability, $s_{0}$ is the sticking probability for etchant species on the free adsorption site, and θ is the fraction of adsorption sites occupied by reaction products, and $\Gamma_{F}\approx 0.25n_{F}\upsilon_{T}$ is the thermal flux of F atoms with the gas-phase density of $n_{F}$. As such, even if the nearly constant surface temperature provides $s_{0}$ ≈ const, $\gamma_{R}$ exhibits the sensitivity to changes in processing conditions through the fraction of free adsorption sites (1−θ).
2) The growth of the fluorocarbon polymer film is provided by non-saturated fluorocarbon species with two and more free bonds [13,14,15,40]. In given gas systems, these are CF_2_ and CF radicals.
3) The decomposition of the fluorocarbon polymer film appears through both physical (sputtering by ion bombardment) and chemical (interaction with oxygen atoms) pathways [13,14,39]. Under the given process conditions (low-oxygenated plasma, constant O_2_ fraction in a feed gas), the relative change in the polymer film thickness may be traced by the parameter $\Gamma_{pol}/\sqrt{\varepsilon_{i}}\Gamma_{+}$, where $\Gamma_{pol}=\;\Gamma_{CF_{2}}+\Gamma_{CF}$ is the total flux of polymerizing species coming from a gas phase.

## 3. Results and Discussion

### 3.1. Etching Rates and Phenomenological Etching Kinetics

**Figure 1:**
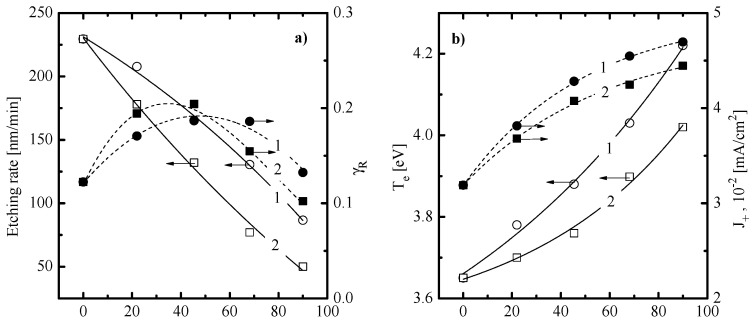

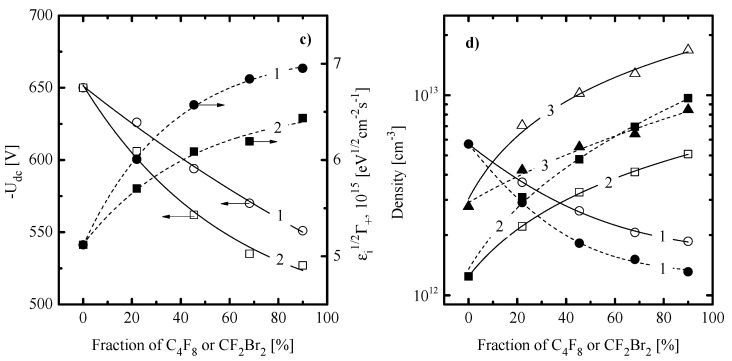
Effect of gas mixing ratios on SiO_x_N_y_ etching kinetics (**a**) and plasma characteristics (**b**–**d**) at *p* = 10 mTorr, *W* = 100 W and $y_{O_{2}}$ = 10%: (**a**) etching rate and effective reaction probability; (**b**) electron temperature and ion current density; (**c**) negative dc bias and the parameter $\sqrt{\varepsilon_{i}}\Gamma_{+}$ characterizing ion energy flux; and (**d**) atoms and radical densities. In (**a**–**c**), numerical labels on curves relate to CF_4_ + C_4_F_8_ + O_2_ (1) or CF_4_ + CF_2_Br_2_ + O_2_ (2) plasmas. In Figure (**d**), labels on curves relate to F (1), CF (2) and CF2 (3) species in CF_4_ + C_4_F_8_ + O_2_ (solid lines) or CF_4_ + CF_2_Br_2_ + O_2_ (dashed lines).

**Figure 2:**
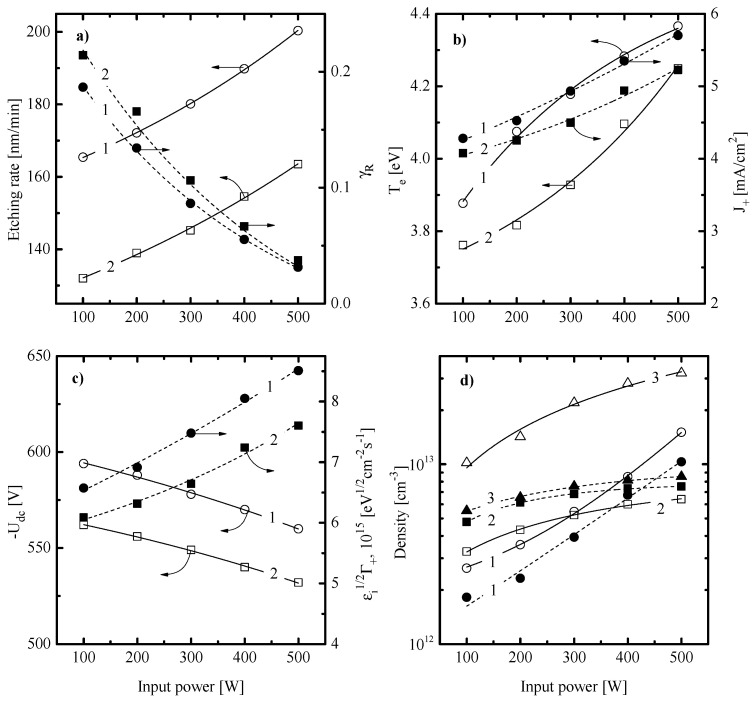
Effect of input power on SiO_x_N_y_ etching kinetics (**a**) and plasma characteristics (**b**–**d**) at *p* = 10 mTorr for 45% CF_4_ + 45% C_4_F_8_ (CF_2_Br_2_) + 10% O_2_ gas mixtures: %: (**a**) etching rate and effective reaction probability; (**b**) electron temperature and ion current density; (**c**) negative dc bias and the parameter $\sqrt{\varepsilon_{i}}\Gamma_{+}$ characterizing ion energy flux; and (**d**) atoms and radical densities. Numerical labels on curves are as in Figure 1.

**Figure 3:**
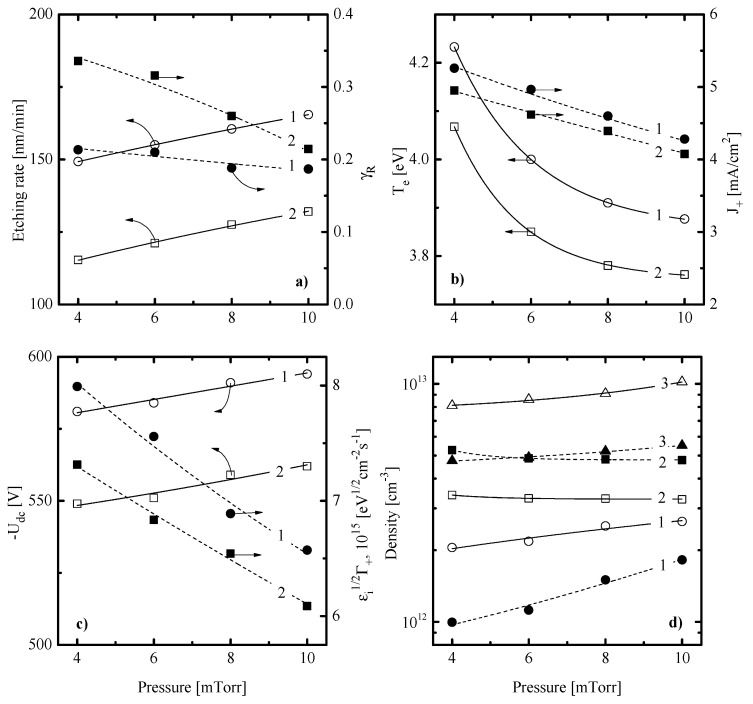
Effect of gas pressure on SiO_x_N_y_ etching kinetics (**a**) and plasma characteristics (**b–d**) at *W* = 100 W for 45% CF_4_ + 45% C_4_F_8_ (CF_2_Br_2_) + 10% O_2_ gas mixtures: %: (**a**) etching rate and effective reaction probability; (**b**) electron temperature and ion current density; (**c**) negative dc bias and the parameter $\sqrt{\varepsilon_{i}}\Gamma_{+}$ characterizing ion energy flux; and (**d**) atoms and radical densities. Numerical labels on curves are as in Figure 1.

Figure 1a, Figure 2a and Figure 3a illustrate how the SiO_x_N_y_ etching rate depends on CF_4_/X (X = C_4_F_8_ or CF_2_Br_2_) mixing ratio, input power and gas pressure. When analyzing these data while accounting for the previous etching experience of silicon-based materials in fluorocarbon gas plasmas [2,3,41,42,43], some preliminary conclusions concerning SiO_x_N_y_ etching kinetics and etching mechanisms can be made. First, similar behaviors of SiO_x_N_y_ etching rates for the cases of X = C_4_F_8_ and CF_2_Br_2_ probably mean that etching processes in both gas mixtures appear in one and the same etching regime and are driven by one and the same active species. In fact, this points to principally identical etching mechanisms as well as similar process optimization algorithms through variations in input plasma parameters. Second, an increase in SiO_x_N_y_ etching rate vs. input power (Figure 2a) and gas pressure (Figure 3a) looks quite typical for the reaction-rate-limited etching regime which is controlled by the gas-phase density and flux of fluorine atoms. As such, one can assume the ion-assisted heterogeneous chemical reaction Si + xF → SiFx to be the process-limiting stage as well as neglect the impact from the side of Br atoms in the case of X = CF_2_Br_2_. The last suggestion becomes reasonable when taking into account the much lower reaction probability in the Si/Br system compared with the Si/F one [44]. Such effect is normally attributed to the bigger size of a Br atom that retards its penetration inside the lattice and leads to the formation of low volatile non-saturated SiBr_x_ compounds [44,45,46]. Thirdly, within the above etching mechanism, a decrease in SiO_x_N_y_ etching rates toward C_4_F_8_- and CF_2_Br_2_-rich plasmas (Figure 1a) may result from corresponding behaviors of F atom fluxes. In the case of X = C_4_F_8_, such asserting is in good agreement with previously published data for CF_4_- and C_4_F_8_-based plasmas which were obtained using the combination of plasma diagnostics and modeling tools. Particularly, Chun et al. [47] reported a lower F atom density in the weakly oxygenated C_4_F_8_ + O_2_ + Ar plasma compared with CF_4_ + O_2_ + Ar one under one and the same operating conditions. In addition, Refs. [35,38] directly demonstrated a decrease in F atoms density when substituting the CF_4_ for C_4_F_8_ in the CF_4_ + C_4_F_8_ + Ar gas mixture. Such a situation is provided by an increase in F atom decay rate through their interaction with C_2_F_4_ species [35,38].

### 3.2. Plasma Parameters and Densities of Active Species

In order to obtain more details concerning the SiO_x_N_y_ etching mechanism, the data on both electron (electron temperature, electron density) an ion (ion flux, ion bombardment energy) related plasma parameters as well as on fluxes of neutral active species (F atoms, polymerizing radicals) are required. For this purpose, we applied plasma diagnostics by Langmuir probes and quadrupole mass-spectrometry. Results represented in Figure 1b,c, Figure 2b,c and Figure 3b,c may be commented on as follows:

- An increase in $T_{e}$ towards higher fractions of X gas (3.6–4.2 eV for X = C_4_F_8_ vs. 3.6–4.0 eV for X = CF_2_Br_2_ at *p* = 10 mTorr and W = 100 W, see Figure 1b) obviously points out on decreasing electron energy losses in inelastic processes with dominant neutral species. A similar effect has been reported for the CF_4_ + C_4_F_8_ + Ar gas mixture after the substitution of CF_4_ for C_4_F_8_ [35,38]. Accordingly, the growth of $n_{+}$ and $J_{+}$ (0.032–0.047 mA/cm^2^ for X = C_4_F_8_ vs. 0.032–0.044 mA/cm^2^ for X = CF_2_Br_2_ at *p* = 10 mTorr and W = 100 W, see Figure 1b) in the case of X = C_4_F_8_ may be surely associated with (a) increasing ionization rate coefficients for all neutral species; (b) increasing numbers of particles with lower ionization thresholds (since the dominant gas-phase component changes from CF_4_ and F in CF_4_-rich plasmas to CF_2_ and CF in C_4_F_8_-rich plasmas, as shown in Refs. [35,38]); and c) lower electronegativity of the C_4_F_8_ plasma compared with CF_4_ one [47,48,49]. The latter means that a decrease in the CF_4_/C_4_F_8_ mixing ratio retards the decay of positive ions and electrons through ion–ion recombination and dissociative attachment, respectively. Probably, similar mechanisms also do work in the case of X = CF_2_Br_2_. An indirect proof is the similar changes in densities of F atoms and CF_x_ (x = 1, 2) radicals in both gas systems (Figure 1d). From Figure 1c, it can be seen that, in both gas mixtures, a decrease in $-U_{dc}$ (650–551 V for X = C_4_F_8_ vs. 650–527 V for X = CF_2_B_r2_ at p = 10 mTorr and W = 100 W, see Figure 1c) does not compensate for increasing ion flux. As a result, one can obtain increasing efficiency of the physical etching pathway, as follows from the change in $\sqrt{\varepsilon_{i}}\Gamma_{+}$ (Figure 1c).
- An increase in $T_{e}$ toward higher input powers (3.9–4.4 eV for X = C_4_F_8_ vs. 3.8–4.3 eV for X = CF_2_Br_2_ at 50% of X gas and p = 10 mTorr, see Figure 2b) probably results from a decrease in electron energy losses for vibrational and electronic excitations. This effect is provided by increasing electron-impact dissociation rates for multi-atomic components which enriches the gas phase by less saturated radicals and atomic species. Accordingly, the monotonic increase in $J_{+}$ (0.043–0.057 mA/cm^2^ for X = C_4_F_8_ vs. 0.041–0.052 mA/cm^2^ for X = CF_2_Br_2_ at 50% of X gas and p = 10 mTorr, see Figure 2b) is due to same changes in total ionization rates which are accelerated by the growth of electron density. The corresponding relationship between W and $n_{e}$ may be easily traced through the input power balance equation [20]. In addition, the growth of ion flux overlaps the weak decrease in $-U_{dc}$ (Figure 2c) and causes an intensification of the physical etching pathway ($\sqrt{\varepsilon_{i}}\Gamma_{+}\;$= 6.6 × 10^15^–8.7 × 10^15^ eV^1/2^cm^−2^s^−1^ for X = C_4_F_8_ vs. 6.1 × 10^15^–7.6 × 10^15^ eV^1/2^cm^−2^s^−1^ for X = CF_2_Br_2_ at 50% of X gas and p = 10 mTorr, see Figure 2c). Such situation is quite typical for many fluorocarbon-based plasmas under the conventional reactive-ion etching conditions [41,42,43].
- A decrease in $T_{e}$ toward higher pressures (4.2–3.9 eV for X = C_4_F_8_ vs. 4.1–3.8 eV for X = CF_2_Br_2_ at 50% of X gas and W = 100 W, see Figure 3b) is due to an increase in both electron-neutral collision frequency and the overall electron energy loss. A similar tendency for $J_{+}$ (0.053–0.043 mA/cm^2^ for X = C_4_F_8_ vs. 0.050–0.041 mA/cm^2^ for X = CF_2_Br_2_ at 50% of X gas and W = 100 W, see Figure 3b) mainly follow the behavior of $n_{+}$. The latter is suppressed by decreasing ionization rate coefficients (due to the change in $T_{e}$) as well as by increasing ion loss rates (due to increasing plasma electronegativity and negative ion density). Accordingly, the combination of decreasing ion flux and the nearly constant $-U_{dc}$ retards the physical etching pathway at the high pressure end (Figure 3c). Similar effects have been repeatedly reported for various gas systems [2,41,42,43].

When summarizing the above data, one can conclude that both gas mixtures are very similar with respect to electron-impact kinetics and ion-related plasma parameters. Although the case of X = CF_2_Br_2_ is featured by the systematically lower plasma density and ion energy flux, the maximum gap between corresponding values for 90% C_4_F_8_ + 10% O_2_ and 90% CF_2_Br_2_ + 10% O_2_ gas mixtures is below 10%. As such, one can suggest the almost identical efficiencies of physical etching pathways (sputtering of target surface, desorption of low volatile reaction products and decomposition of fluorocarbon polymer film) as well as attribute differences in densities of neutral species to chemical properties of original C_4_F_8_ and CF_2_Br_2_ molecules. It is important to note that the low input power etching regime keeps most principal features of the conventional reactive-ion etching process. This conclusion follows from similar tendencies in both electron- and ion-related plasma parameters with those from Refs. [35,38] as functions of the CF_4_/C_4_F_8_ mixing ratio.

**Figure 4:**
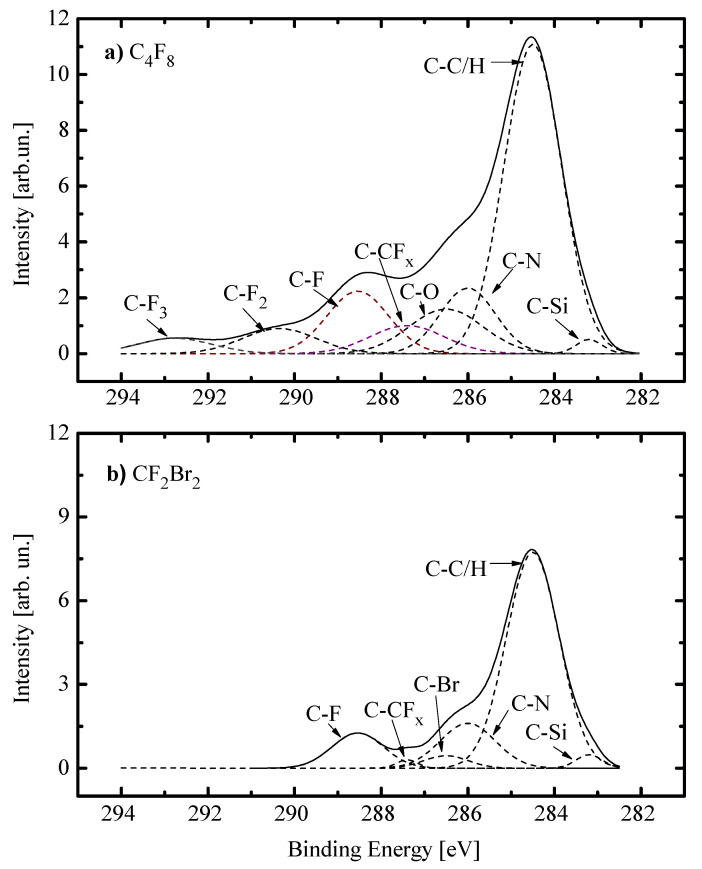
XPS spectra for C 1s on the plasma-treated SiO_x_N_y_ surface after pure C_4_F_8_ (**a**) and CF_2_Br_2_ (**b**) gases. Plasma parameters correspond to Figure 1.

Figure 1d, Figure 2d and Figure 3d illustrate the influence of processing conditions on steady-state densities of selected neural species. From Figure 1d, it can be seen that an increase in the fraction of X gas lowers the F atom density and increases the density of CF_x_ (x = 1, 2) radicals. In the case of X = C_4_F_8_, the behaviors of $n_{CF_{2}}$ and $n_{CF}$ completely correspond to those obtained in our previous works by plasma modeling [35,38]. Particularly, the growth of $n_{CF_{2}}$ results from the corresponding change in their formation rate due to R1: C_4_F_8_ + e → C_3_F_6_ + CF_2_ + e ($k_{1}$ ~ 1.1 × 10−9 cm^3^/s at $T_{e}$ = 4 eV) and R2: C_2_F_4_ + e → 2CF_2_ + e ($k_{2}$ ~ 4.8 × 10−9 cm^3^/s at $T_{e}$ = 4 eV) that follows after R3: C_4_F_8_ + e → 2C_2_F_4_ + e ($k_{3}$ ~ 1.0 × 10−8 cm^3^/s at $T_{e}$ = 4 eV). In the CF_4_ plasma, the formation of CF_2_ radicals is the consequent two-step process (such as R4: CF_4_ + e → CF_3_ + F + e and R5: CF_3_ + e → CF_2_ + F + e) that is limited by the relatively slow R4 ($k_{4}$ ~ 5.0 × 10−10 cm^3^/s at $T_{e}$ = 4 eV). Accordingly, the density of CF always traces that for CF_2_, as these are linked through R6: CF_2_ + e → CF + F + e [38,47,48]. Finally, decreasing density of fluorine atoms is due to their effective decay in the gas-phase process R7: F + C_2_F_4_ → CF_2_ + CF_3_ ($k_{7}$ ~ 10–11 cm^3^/s) [38,47]. In the case of X = CF_2_Br_2_, the similar situation may take place only if (a) the CF_2_Br_2_ is the better source of CF_2_ radicals compared with CF_4_; and (b) the CF_2_Br_2_ is the worse source of F atoms and/or provides their additional decay channel, by analogy with R7. From Ref. [19], it can be understood that the dissociation energy for the C-Br bond (~318 kJ/mol) is much lower compared with the C-F one (~514 kJ/mol). That is why one can imagine the dominant dissociation pathway for CF_2_Br_2_ molecules as the consequent reaction mechanism in a form of R8: CF_2_Br_2_ + e → CF_2_Br + Br + e and R9: CF_2_Br + e → CF_2_ + Br + e. The evident proof is that, according to our QMS data, the condition $n_{Br}$ > $n_{F}$ surely takes place when the fraction of CF_2_Br_2_ exceeds 40%. Thought R8-R9 is also the two-step process in respect to the formation of CF_2_ radicals, it may be more effective compared with the couple of R4-R5. Reasons are the lower threshold energy, the bigger process cross-section (due to the bigger parent particle size) as well as the acceleration of electron-impact kinetics toward CF_2_Br_2_-rich plasmas, as follows from Figure 1b. Another important feature is the high recombination probability for Br atoms which results in the effective formation of Br_2_ molecules even if those are absent in a feed gas [50,51]. As such, the reaction R10: F + Br_2_ → BrF + Br ($k_{10}$ ~ 2.0 × 10^−10^ cm^3^/s [52]) may easily play the role of R7 to decrease the density of F atoms with increasing fraction of CF_2_Br_2_. Finally, the comparison of two gas systems in the quantitative scale allows one to conclude that:

1) The case of X = CF_2_Br_2_ provides the systematically lower (by ~30%) density of F atoms that corresponds to lower flux of these species to the etched surface and thus, to the lower etching performance.
2) The case of X = C_4_F_8_ provides a systematically higher density of polymerizing CF_x_ (x = 1, 2) radicals which corresponds to higher polymerizing flux and thus, higher deposition rate for the fluorocarbon polymer film. The last fact is supported by direct experiment confirmation after the XPS analysis of the plasma-treated surfaces. Particularly, Figure 4 shows that the treatment in 90% C_4_F_8_ + 10% O_2_ plasma produces much more residual CF_x_ compounds than that for the 90% C_4_F_8_ CF_2_Br_2_ + 10% O_2_ gas system. Since O_2_ content and ion energy flux in both gas systems are rather close, such sufficient difference in surface conditions may be related only to different polymerizing fluxes coming from a gas phase.

### 3.3. Etching Mechanism and Profile Features

**Figure 5:**
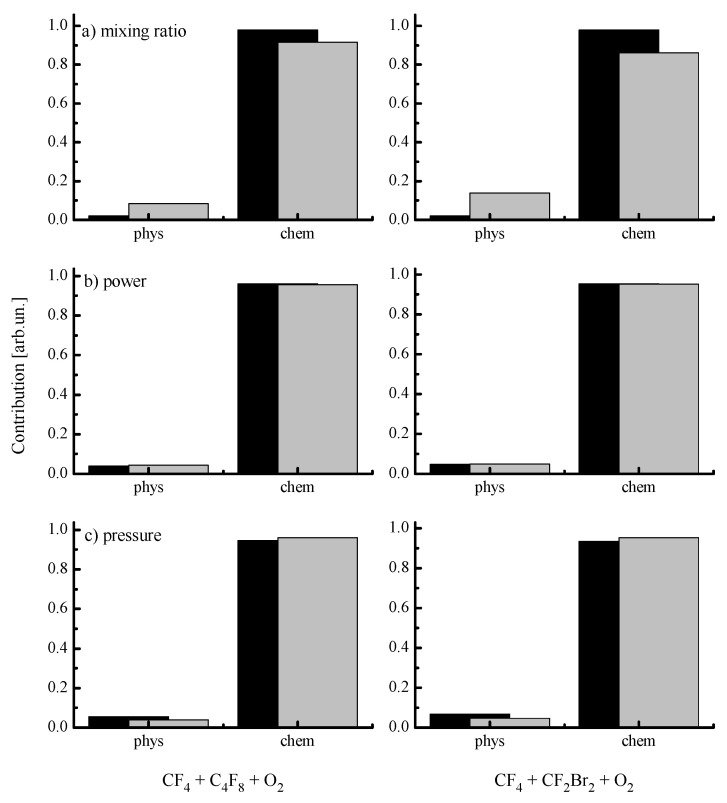
Relative contributions of physical and chemical etching pathways to the SiO_x_N_y_ etching rate. Black and gray bars in (**a**–**c**) correspond to lowest and highest values of variable parameters from Figure 1, Figure 2 and Figure 3, respectively.

From Figure 1 and Figure 3, it can be seen that the behavior of SiO_x_N_y_ etching rate vs. CF_4_/X (X = C_4_F_8_ or CF_2_Br_2_) mixing ratio and gas pressure contradicts that for the ion energy flux (since the latter is traced by the parameter $\sqrt{\varepsilon_{i}}\Gamma_{+}$) while following the change in the F atom flux. Such a situation obviously confirms the earlier made suggestion on the reaction-rate-limited etching regimes in both gas systems under the given set of operating conditions. Another important conclusion that follows from the above data is that $R_{chem}$ >> $R_{phys}$ and R ≈ $R_{chem}$. In order to evaluate the real contribution of $R_{phys}$ to the measured etching rate, one can refer for indirect data on SiO_x_N_y_ sputtering yields [53,54]. From this work, one can conclude that the ion bombardment energy of ~600 eV provides $Y_{S}$~1.2 atom/ion. For given process conditions, the latter may be assumed to be a constant because of rather weak changes in both $-U_{dc}$ (see Figure 1c, Figure 2c and Figure 3c) and $\sqrt{\varepsilon_{i}}$. Therefore, as the fraction of additive X gas changes from 0–90%, $R_{phys}$ occupies the ranges of 2.4 × 10^14^–3.5 × 10^14^ cm^−2^c^−1^ (4.9–7.3 nm/min) for X = C_4_F_8_ and 2.4 × 10^14^–3.5 × 10^14^ cm^−2^c^−1^ (4.9–6.7 nm/min) for X = CF_2_Br_2_. As such, the maximum contribution of $R_{phys}$ to the total SiO_x_N_y_ etching rate slightly exceeds 10% as well as remains below 10% with variations in input power and has pressure (Figure 5). Effective reaction probabilities calculated as $\gamma_{R}=R_{chem}/\Gamma_{F}$, where $R_{chem}=R-R_{phys}$, exhibit process-condition-dependent natures as well as show similar behaviors for both gas mixtures (see Figure 1a, Figure 2a and Figure 3a). The analysis of these data in connection with fluxes of plasma active species allows us to suggest some common features characterizing the SiO_x_N_y_ etching mechanism:

1) The non-monotonic change in $\gamma_{R}$ with increasing CF_4_/X (X = C_4_F_8_ or CF_2_Br_2_) mixing ratio (Figure 1a) may be associated with the transition between thin- and thick-fluorocarbon film etching regimes [13,14,15]. In the case of $y_{X}$ < $y_{CF_{4}}$ (where y are the fractions of corresponding components in a feed gas), one probably obtains either the non-continuous or the thin continuous fluorocarbon film which did not retard the access of F atoms to the etched surface. That is why the behavior of $\gamma_{R}$ follows the increasing ion energy fluxes that accelerated the chemical reaction through the destruction of Si-O and Si-N bonds. Oppositely, the case of $y_{X}$ > $y_{CF_{4}}$ probably corresponds to the thick continuous fluorocarbon film which reduces the flux of F atoms at the film/SiO_x_N_y_ interface. As a result, a decrease in $\gamma_{R}$ is due to an increase in the film thickness, as indicated by the change in the parameter $\Gamma_{pol}/\sqrt{\varepsilon_{i}}\Gamma_{+}$ (8.2–35.5 eV^−1/2^ for 0–90% C_4_F_8_ and 8.2–27.3 eV^−1/2^ for 0–90% CF_2_Br_2_).
2) A decrease in $\gamma_{R}$ towards higher input powers (Figure 2a) under the condition of $y_{X}$ = $y_{CF_{4}}$ correlates with the change in $\Gamma_{pol}/\sqrt{\varepsilon_{i}}\Gamma_{+}$ ratio (21.0–57.1 eV^−1/2^ for 100–500 W at 45% C_4_F_8_ and 18.7–28.8 eV^−1/2^ for 100–500 W at 45% CF_2_Br_2_) and thus, with the fluorocarbon film thickness. This is because an increase in densities and fluxes for CF_x_ (x = 1, 2) species appears to faster compared with the ion energy flux. Obviously, the latter is suppressed by decreasing $-U_{dc}$ and ion bombardment energy.
3) A decrease in $\gamma_{R}$ towards higher gas pressures (Figure 3a) under the condition of $y_{X}$ = $y_{CF_{4}}$ is also in agreement with increasing thickness of the fluorocarbon polymer film, as follows from the change in $\Gamma_{pol}/\sqrt{\varepsilon_{i}}\Gamma_{+}$ ratio (15.0–21.0 eV^−1/2^ for 4–10 mTorr at 45% C_4_F_8_ and 14.9–18.5 eV^−1/2^ for 4–10 mTorr at 45% CF_2_Br_2_). In this case, the growth of polymer film thickness is provided by a combination of increasing flux of polymerizing species and decreasing ion energy flux due to $\Gamma_{+}$. Obviously, the latter is suppressed by both decreasing ionization rate coefficients and ion Bohm velocity.

From above data, one can conclude that SiO_x_N_y_ etching mechanisms in CF_4_ + O_2_ + X (X = C_4_F_8_ or CF_2_Br_2_) plasmas exhibit many common features. The most important among these are: (a) the dominant role of the ion-assisted chemical reaction; (b) the neutral-flux-limited etching regime; and (c) the non-constant effective reaction probability that depends on the fluorocarbon polymer deposition/removal balance. Since such a situation is quite typical for many reactive-ion etching processes in fluorocarbon gas plasmas [2,41,42,43], the substitution of C_4_F_8_ for CF_2_Br_2_ for dry etching purposes will not have a significant impact on both process control algorithms and results.

**Figure 6:**
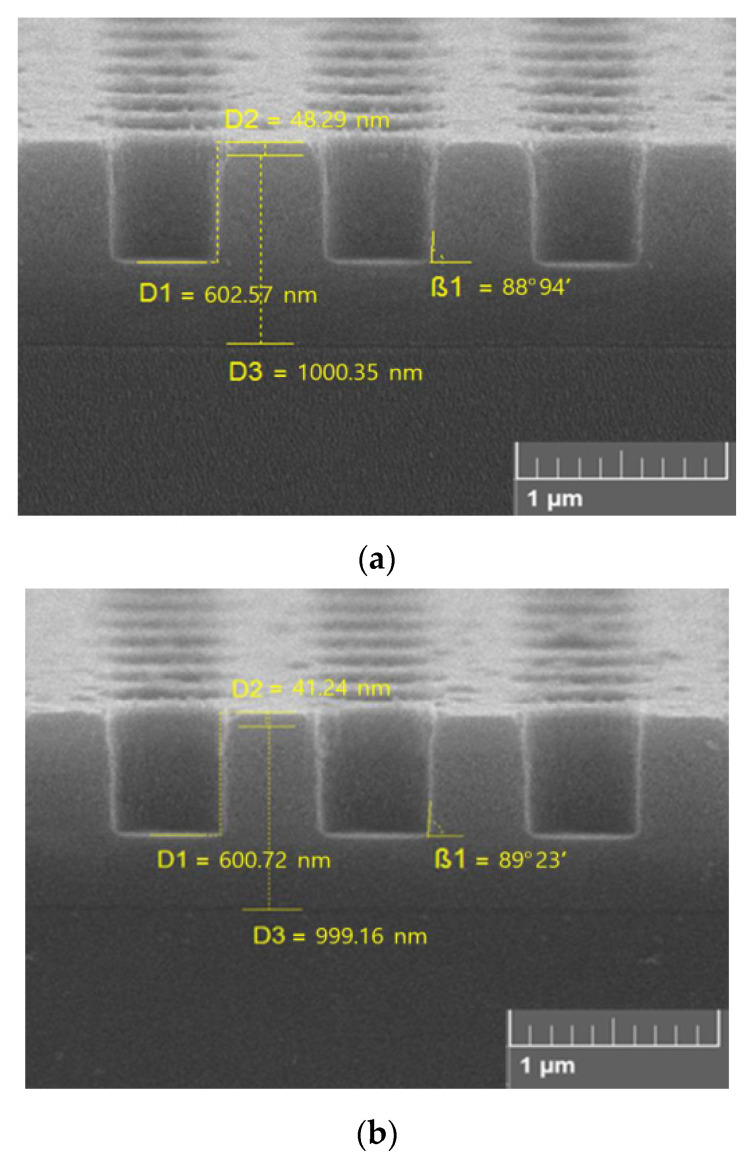
Etched profiles for 45% CF_4_ + 45% C_4_F_8_ + 10% O_2_ (**a**) and 45% CF_4_ + 45% CF_2_Br_2_ + 10% O_2_ (**b**) plasmas. Plasma parameters correspond to Figure 1.

Finally, Figure 6 provides the comparison of etched profiles obtained in CF_4_ + O_2_ + X (X = C_4_F_8_ or CF_2_Br_2_) plasmas. It can be seen that at the etched depth of ~600 nm, profile angles are 88.94° for X = C_4_F_8_ and 89.23° for X = CF_2_Br_2_. Since the difference between these values is quite close to the experimental error, one can only conclude that the use of liquid CF_2_Br_2_ instead of gaseous C_4_F_8_ for the purpose of the given etching process does not degrade the dimensional etching performance. At the same time, the lower polymerization ability of CF_2_Br_2_–containing plasmas (as it clearly follows from data discussed in Section 3.2) reasonably suggests the worse protection of sidewalls against F atoms and scattered ions. Therefore, the comparable etched profile in the case of X = CF_2_Br_2_ may probably be attributed to the lower neutral/charged ratio ($\Gamma_{F}/\sqrt{\varepsilon_{i}}\Gamma_{+}$ = 6.3 eV^−1/2^ for 45% C_4_F_8_ and 4.5 eV^−1/2^ for 45% CF_2_Br_2_).

## 4. Conclusions

In this work, we investigated reactive-ion etching kinetics, etching mechanism and surface conditions for SiO_x_N_y_ films in CF_4_ + O_2_ + X (X = C_4_F_8_ or CF_2_Br_2_) inductively coupled plasmas in a low-power regime. The use of the lower power etching mode was aimed at reducing ion density and flux in order to minimize the degradation of electric properties at plasma-treated surfaces. It was found that: 1) both gas systems are characterized by identical changes in SiO_x_N_y_ etching kinetics with variations in gas pressure, input power and CF_4_/X mixing ratios; and 2) the gas system with X = CF_2_Br_2_ provides lower absolute etching rates, exhibits the weaker contamination of etched surfaces by the fluorocarbon polymer as well as results in a bit more vertical etching profile. In order to understand the above features, the properties of CF_4_ + O_2_ + X (X = C_4_F_8_ or CF_2_Br_2_) plasmas were studied using Langmuir probe diagnostics and quadrupole mass-spectrometry. The corresponding results indicated that: 1) similar behaviors in plasma parameters (electron temperature, ion current density) and fluxes of active species for X = C_4_F_8_ or CF_2_Br_2_; and 2) lower F atom density, density of polymerizing radicals and neutral/charged ratio for X = CF_2_Br_2_. The analysis of the SiO_x_N_y_ etching mechanism suggested the effectiveness of the F atom flux-limited etching regime with the process-condition-dependent effective probability for the SiO_x_N_y_ + F reaction. The latter depends on the fluorocarbon polymer deposition/removal balance.

## References

[1] Sze S.M. (1988). VLSI Technology.

[2] Wolf S., Tauber R.N. (2000). Silicon Processing for the VLSI era.

[3] Sugano T. (1990). Applications of Plasma Processes to VLSI Technology.

[4] Niklasson G.A., Eriksson T.S., Brantervik K. (1989). Dielectric properties of silicon oxynitride films. Appl. Phys. Lett..

[5] Wörhoffz K., Hilderink L.T.H., Driessen A., Lambeck P.V. (2002). Silicon oxynitride: A versatile material for integrated optics applications. J. Electrochem. Soc..

[6] Son J., Efremov A., Chun I., Yeom G.Y., Kwon K.-H. (2014). On the LPCVD-formed SiO_2_ etching mechanism in CF_4_/Ar/O_2_ inductively coupled plasmas: Effects of gas mixing ratios and gas pressure. Plasma Chem. Plasma Proc..

[7] Li X., Ling L., Hua X., Fukasawa M., Oehrlein G.S., Barela M., Anderson H.M. (2003). Effects of Ar and O_2_ additives on SiO_2_ etching in C_4_F_8_-based plasmas. J. Vac. Sci. Technol. A.

[8] Schaepkens M., Standaert T.E.F.M., Rueger N.R., Sebel P.G.M., Oehrlein G.S., Cook J.M. (1999). Study of the SiO_2_-to-Si_3_N_4_ etch selectivity mechanism in inductively coupled fluorocarbon plasmas and a comparison with the SiO_2_-to-Si mechanism. J. Vac. Sci. Technol. A.

[9] Chen L., Xu L., Li D., Lin B. (2009). Mechanism of selective Si_3_N_4_ etching over SiO_2_ in hydrogen-containing fluorocarbon plasma. Microelectron. Eng..

[10] Kastenmeier B.E.E., Matsuo P.J., Beulens J.J., Oehrlein G.S. (1996). Chemical dry etching of silicon nitride and silicon dioxide using CF_4_/O_2_/N_2_ gas mixtures. J. Vac. Sci. Technol. A.

[11] Kastenmeier B.E.E., Matsuo P.J., Oehrlein G.S. (1999). Highly selective etching of silicon nitride over silicon and silicon dioxide. J. Vac. Sci. Technol. A.

[12] Lee H.K., Chung K.S., Yu J.S. (2009). Selective etching of thick Si_3_N_4_, SiO_2_ and Si by using CF_4_/O_2_ and C_2_F_6_ gases with or without O_2_ or ar addition. J. Korean Phys. Soc..

[13] Matsui M., Tatsumi T., Sekine M. (2001). Relationship of etch reaction and reactive species flux in C_4_F_8_/Ar/O_2_ plasma for SiO_2_ selective etching over Si and Si_3_N_4_. J. Vac. Sci. Technol. A.

[14] Lele C., Liang Z., Linda X., Dongxia L., Hui C., Tod P. (2009). Role of CF_2_ in the etching of SiO_2_, Si_3_N_4_ and Si in fluorocarbon plasma. J. Semicond..

[15] Standaert T.E.F.M., Hedlund C., Joseph E.A., Oehrlein G.S., Dalton T.J. (2004). Role of fluorocarbon film formation in the etching of silicon, silicon dioxide, silicon nitride, and amorphous hydrogenated silicon carbide. J. Vac. Sci. Technol. A.

[16] Kim B., Kim J., Lee S.H., Park J., Lee B.T. (2005). Plasma etching of silicon oxynitride in a low-pressure C_2_F_6_ plasma. J. Korean Phys. Soc..

[17] Cavallari C., Gualandris F. (1987). Plasma processing for silicon oxynitride films. J. Electrochem. Soc..

[18] Ueno K., Kikkawa T., Tokashiki K. (1995). Reactive ion etching of silicon oxynitride formed by plasma-enhanced chemical vapor deposition. J. Vac. Sci. Technol. B.

[19] Lide D.R. (1998). Handbook of Chemistry and Physics.

[20] Lieberman M.A., Lichtenberg A.J. (1994). Principles of Plasma Discharges and Materials Processing.

[21] Mogab C.J., Adams A.C., Flamm D.L. (1978). Plasma etching of Si and SiO_2_—The effect of oxygen additions to CF_4_ Plasmas. J. Appl. Phys..

[22] Lee J., Kim J., Efremov A., Kim C., Lee H.W., Kwon K.-H. (2019). Etching mechanisms and surface conditions for SiO_x_N_y_ thin films in CF_4_ + CHF_3_ + O_2_ inductively coupled plasma. Plasma Chem. Plasma Process..

[23] Tran-Quinn T., Lakritz M. (2008). Unsaturated fluorocarbons in the etching process, environmental benefit, technical hurdles. Proceedings of the Conference IEEE/SEMI Advanced Semiconductor Manufacturing Conference.

[24] Kiehlbauch M.W., Graves D.B. (2001). Temperature resolved modeling of plasma abatement of perfluorinated compounds. J. Appl. Phys..

[25] Bolaji B.O., Huan Z. (2013). Ozone depletion and global warming: Case for the use of natural refrigerant–a review. Renew. Sustain. Energ. Rev..

[26] Krishnan N., Smati R., Raoux S., Dornfeld D. Alternatives to reduce perfluorinated compound (PFC) emissions from semiconductor dielectric etch processes: Meeting environmental commitments while minimizing costs. Proceedings of the Conference IEEE International Symposium on Electronics and the Environment.

[27] Mocella M.T. (1996). PFC emission control options for plasma processing tools: A current Assessment. MRS Online Proc. Libr. Arch..

[28] Beppu T., Mitsui Y., Sakai K., Sekiya A. (2002). New alternative gas process feasibility study for PFC emission reduction from semiconductor CVD chamber cleaning. Proceedings of the Greenhouse Gas Control Technologies-6th International Conference.

[29] Li Y.E.D., Paganessi J.E., Rufin D. (2000). Emission reduction of perfluorocompounds in semiconductor manufacturers via capture and recycle. Green Eng..

[30] Tsai W.T., Chen H.P., Hsien W.Y. (2002). A review of uses, environmental hazards and recovery/recycle technologies of perfluorocarbons (PFCs) emissions from the semiconductor manufacturing processes. J. Low. Prevent. Proc..

[31] Hodnebrog Ø., Etminan M., Fuglestvedt J.S., Marston G., Myhre G., Nielsen C.J., Shine K.P., Wallington T.J. (2013). Global warming potentials and radiative efficiencies of halocarbons and related compounds: A comprehensive review. Rev. Geophys..

[32] Xiang W.H. (2001). Vapor pressures, critical parameters, boiling points, and triple points of halomethane molecular substances. J. Phys. Chem. Ref. Data.

[33] Veselov D.S., Bakun A.D., Voronov Y.A. (2016). Reactive ion etching of silicon using low-power plasma etcher. J. Phys. Conf. Ser..

[34] Ashraf M., Sundararajan S.V., Grenc G. (2017). Low-power, low-pressure reactive-ion etching process for silicon etching with vertical and smooth walls for mechanobiology application. J. Micro Nanolith. MEMS MOEMS.

[35] Lee J., Efremov A., Yeom G.Y., Lim N., Kwon K.-H. (2015). Application of Si and SiO_2_ etching mechanisms in CF_4_/C_4_F_8_/Ar inductively coupled plasmas for nanoscale patterns. J. Nanosci. Nanotechnol..

[36] Johnson E.O., Malter L. (1950). A floating double probe method for measurements in gas discharges. Phys. Rev..

[37] Shun’ko E.V. (2008). Langmuir Probe in Theory and Practice.

[38] Lee J., Efremov A., Kwon K.-H. (2018). On the relationships between plasma chemistry, etching kinetics and etching residues in CF_4_+C_4_F_8_+Ar and CF_4_+CH_2_F_2_+ Ar plasmas with various CF_4_/C_4_F_8_ and CF_4_/CH_2_F_2_ mixing ratios. Vacuum.

[39] Gray D.C., Tepermeister I., Sawin H.H. (1993). Phenomenological modeling of ion-enhanced surface kinetics in fluorine-based plasma etching. J. Vac. Sci. Technol. B.

[40] Stoffels W.W., Stoffels E., Tachibana K. (1998). Polymerization of fluorocarbons in reactive ion etching plasmas. J. Vac. Sci. Technol. A.

[41] Winters H.F., Coburn J.W., Chuang T.J. (1983). Surface processes in plasma-assisted etching environments. J. Vac. Sci. Technol. B.

[42] Coburn J.W. (1982). Plasma Etching and Reactive Ion Etching, AVS Monograph Series.

[43] Roosmalen A.J., Baggerman J.A.G., Brader S.J.H. (1991). Dry Etching For VLSI.

[44] Vitale S.A., Chae H., Sawin H.H. (2001). Silicon etching yields in F_2_, Cl_2_, Br_2_, and HBr high density plasmas. J. Vac. Sci. Technol. A.

[45] Belen R.J., Gomez S., Kiehlbauch M., Aydil E.S. (2006). Feature scale model of Si etching in SF_6_/O_2_/HBr plasma and comparison with experiments. J. Vac. Sci. Technol. A.

[46] Bestwick T.D., Oehrlane G.S. (1990). Reactive ion etching of silicon using bromine containing plasmas. J. Vac. Sci. Technol. A.

[47] Chun I., Efremov A., Yeom G.Y., Kwon K.-H. (2015). A comparative study of CF_4_/O_2_/Ar and C_4_F_8_/O_2_/Ar plasmas for dry etching applications. Thin Solid Film.

[48] Rauf S., Ventzek P.L. (2012). Model for an inductively coupled Ar/c-C_4_F_8_ plasma discharge. J. Vac. Sci. Technol. A.

[49] Kimura T., Noto M. (2006). Experimental study and global model of inductively coupled CF_4_/O_2_ discharges. J. Appl. Phys..

[50] Efremov A., Lee J., Kwon K.-H. (2017). A comparative study of CF_4_, Cl_2_ and HBr + Ar inductively coupled plasmas for dry etching applications. Thin Solid Film.

[51] Kwon K.-H., Efremov A., Kim M., Min N.K., Jeong J., Kim K. (2010). A model-based analysis of plasma parameters and composition in HBr/X (X=Ar, He, N_2_) inductively coupled plasmas. J. Electrochem. Soc..

[52] NIST Chemical Kinetics Database. https://kinetics.nist.gov/kinetics/index.jsp.

[53] Kim D.-H., Lee G.-H., Lee S.Y., Kim D.H. (2006). Atomic scale simulation of physical sputtering of silicon oxide and silicon nitride thin films. J. Cryst. Growth.

[54] Seah M.P., Nunney T.S. (2010). Sputtering yields of compounds using argon ions. J. Phys. D Appl. Phys..

